# Artificial Dilatation of the Uterine Cervix during Labor

**Published:** 1886-03-20

**Authors:** W. R. Chittick

**Affiliations:** Detroit, Michigan


### ARTIFICIAL DILATATION OF THE UTERINE CERVIX DURING LABOR.

By Dr. W. R. Chittick, Detroit, Mich.

**Read before the Detroit Obstetrical and Gynecological Society.**

Artificial dilatation of the uterine cervix, in cases where it may be practiced and where it is called for, is, in my opinion, a justifiable and worthy procedure, provided the operator understands just how to go about it. There are many cases of labor in which by judicious and reasonably prompt action on the part of the attending physician, much time and suffering may be saved the patient, if he chooses to practice this procedure.

I know many specialists are opposed to the practice of this operation and usually condemn it when opportunity, is offered them to do so. Why they do so I cannot understand when they advocate operations and procedures that are fully as open to criticism, and are, in the minds of many general practitioners, looked upon with an unapproving eye.

This is a day of civilization, and consequently one of many artificial procedures. We cannot expect to find all women now-a-days having normal labors; if we were all in a state of nature it would be different, but even then there would occasionally be dffi-culties. The obstetrician of to-day must be armed with ergot, forceps, opiates and sometimes chloroform, and is not always slow to use one or more of them—especially the ergot.

Why then should we not practice a procedure that is advocated by some of our best practitioners—the more especially as it is one calculated to shorten the period of labor? The majority of confinements are severe enough; long hours of suffering by mothers who, if they could, would soon shorten the time, and who many times are tempted to, and often do, take means to terminate the life of the fœtus, thinking that by that means they will lessen their sufferings as well as get rid of the trouble and care of a child.

If we can by any legitimate means shorten the time of child-birth I think we certainly ought to do so. When our patients call us in they expect to get some help from us, and if we educate and train ourselves to give them the greatest amount of relief that science and art can devise, then we will perform the functions of true physicians.

There is another reason why we should make child-birth as easy and as short as our knowledge and skill will permit, and it is that it will serve to make it less dreaded and encourage more wives to become mothers. Then again it will tend to lessen the number of miscarriages and abortions that are so common. It has struck me as a fact that criminal abortions are more common among the more intelligent women than among the so-called lower classes. So, if we can do anything to make the confinements of these women less painful in bringing into the world children who, with the advantages they would have, ought to make better men and women, and, therefore, add to the list of reputable citizens, we will accomplish a good thing.

In regard to the operation itself: As is now well understood, the dilatation of the os is produced by the contraction of the longitudinal fibres of the uterus, which are more numerous, longer and stronger than the circular fibres of the mouth of the womb. The contractions, as a whole, also serve to diminish the size of the womb, and thereby press upon its contents and tend to push out the entire ovum. The fœtus being surrounded by the liquor amnii does not directly act on the os, but the bag of waters becoming tense, offers something for the muscular fibres to pull over, and, being pliable, to protrude through the opening made. Now, on a little thought, it must appear clear to every one that the result of the whole process is simply to open wide the mouth of the womb, mere mechanical dilatation, in order to allow it to expel its contents. Now, the first stage of labor is often protracted much longer than there is any necessity for, and by artificial dilatation we can shorten it from one to six hours.

There are two methods of artificially dilating the os: first, by digital manipulation; and, second, by using hydrostatic bags. I have had no experience with the bags, but can say that I have had excellent results from dilating the os with the fingers. I have had cases in which there seemed to be but very little dilatation produced by the natural means, in which, as soon as I applied my fingers, the pains seemed to take on a renewed vigor, and labor progressed happily. I had one such case that remained stationary from the middle of the night until 11 o'clock the next day, when I saw her for the first time, that I delivered within half hour after first applying my fingers. Everything was ready for dilatation, but for some reason it did not progress.

I think that *gentle* manipulations are the best. There is no necessity whatever for the use of force, as the manipulations are simply expected to produce stimulation, and this exciting the uterus to renewed vigor, make the pains stronger—sometimes very strong. All that is necessary is to insert the first two fingers of either hand into the os *between* pains, and to gently widen them and run them around between the os and the membranes, especially at the anterior portion. Force should not be used because it is not necessary, and might prove injurious. Neither should the operation be hastened, for nothing is to be gained by it. —*Medical Age.*

